# Evaluation of acute skin irritation and phototoxicity by aqueous and ethanol fractions of *Angelica keiskei*

**DOI:** 10.3892/etm.2012.782

**Published:** 2012-10-30

**Authors:** SANG-HAN LEE

**Affiliations:** Department of Food Science and Biotechnology and Food and Bio-Industry Research Institute, Kyungpook National University, Daegu 702-701, Republic of Korea

**Keywords:** *Angelica keiskei*, skin irritancy, phototoxicity, fraction

## Abstract

In this study, to assess whether aqueous and ethanol fractions of *Angelica keiskei* induce acute skin irritation and phototoxicity, acute skin irritancy and phototoxicity tests were performed. The skin of rabbits or guinea pigs was treated with these fractions (100 mg/dose) and whether the animals sustained significant skin damage was determined. The data demonstrated that the aqueous and ethanol fractions of *Angelica keiskei* did not induce acute toxicity in the skin of the animals, as assessed by anatomical and pathological observations. The results from the present study suggest that these aqueous and ethanol fractions of *Angelica keiskei* have promising potential uses as cosmetic ingredients that do not induce significant levels of skin irritation or phototoxicity.

## Introduction

It is well known that many medicinal plants are beneficial sources of minerals, vitamins, dietary fiber and various phytochemicals (1). Certain herbs are popular at present since their ingredients may not only regulate body homeostasis but also prevent several degenerative diseases (2,3). More than 60 species of the genus *Angelica* are established plant sources of vitamin B complexes, vitamin C, chlorophylls and minerals (4). The highly potent antioxidant properties of the fresh leaves make these functional food ingredients. Additionally, members of this group of plants have traditionally been used as anti-inflammatory agents, as well as remedies for colds, flu, hepatitis, arthritis, indigestion, coughing, chronic bronchitis, fever, cancer and bacterial infections (5–7) due to the group’s flavonoid, saponin and coumarin content. Further studies have revealed that oils from these plants are able to inhibit the growth of PANC-1 pancreatic cancer cells (8).

*Angelica keiskei* has been widely used as an alternative medicine for treating irritable bowel syndrome, arthritis and immune diseases (9). It has also been demonstrated that this plant reduces inflammation *in vivo* in a chronic ethanol-induced test (10). The administration of *Angelica keiskei* extracts to ICR mice at 10, 25 and 50 mg/kg *per os* (p.o.; by mouth) improves alcohol-induced hepatotoxicity, suggesting that these extracts indirectly protect the liver against free radical attack (10). However, limited scientific information has prevented the use of *Angelica keiskei* for treating various degenerative disorders. Previously, the anti-asthmatic activities of an aqueous extract in an ovalbumin-induced animal model was investigated in our laboratory (11). *Angelica* extract was orally administered to ovalbumin-sensitized mice and their lungs were analyzed to compare IL-4 and IL-13 cytokine expression levels in the tissues using immunohistochemistry. The extract was revealed to have potent anti-asthmatic effects capable of controlling CD4^+^ cell populations, IL-4 and IL-13 expression and asthma-associated biomarkers in the lungs (11). Previously, several alkylated chalcones obtained from *Angelica* have been observed to inhibit influenza virus neuraminidase (12). Other preventive approaches against various degenerative diseases may ameliorate the opportunistic damage and/or causes (13,14).

The Korea Food and Drug Administration (KFDA) has suggested that guidelines (*in vitro* 3T3 NRU phototoxicity test and local lymph node assay) should be established for evaluating functional cosmetic ingredients [http://www.kfda.go.kr/search/search/search.kfda (In Korean)]. Plant extracts with pungent scents appear to cause skin irritation. Unwanted reactions to cosmetics are common in patients with allergic dermatitis (15). Since various side-effects may be caused by acute or chronic toxicity, irritation or sensitization, various *in vivo* animal models, as well as *in vitro*, semi *in vivo* and *ex vivo* models, should be used in further toxicity studies, although they are modified tests (16). If a cosmetic component or constituent is demonstrated to be non-toxic to the skin in animals or clinical trials, its use should be approved. Although cosmetic ingredients have rarely caused serious damage, no studies have conclusively demonstrated that these substances actively protect the skin or promote tissue regeneration.

Previously, our group published the results of a study demonstrating the effects of fractions of the plant *Angelica keiskei* on eye mucosa irritancy (17). In the present study, acute skin irritation and phototoxicity tests were performed using animal models to analyze the *in vivo* effects of the *Angelica keiskei* leaf. Various parameters were measured by comparing the acute toxicity tests with calculated degrees to ascertain whether the *Angelica* extracts may potentially be used for cosmetic applications without damaging the skin.

## Materials and methods

### Animal care and use

New Zealand white (NZW) rabbits (9-week-old males weighing between 2.1 and 2.4 kg) and guinea pigs (Hartley, 7-week-old males weighing between 319.6 and 372.9 g) were purchased from Samtaco Korea (Osan, Korea) and used for the skin irritancy and phototoxicity tests, respectively. The animals were fed a commercial diet (Purina Korea, Seoul, Korea) and water *ad libitum* throughout all the experiments. The study protocols complied with the guidelines of the International Association for the Study of Pain Committee for Research and Ethical Issues (18) and strictly observed the internal guidelines of the University Animal Ethics Committee. All animals were acclimated to the laboratory environment for at least one week prior to the commencement of the experiments.

### Sample preparation

*Angelica keiskei* leaves purchased from Myung-il Farm Co. (Eumsung, Korea) were used throughout the experiments. Sample preparation was carried out as previously described (19). The slice-dried leaves were pulverized with a homogenizer (20,000 rpm for 15 min; Shin-Il, Seoul, Korea) to obtain aqueous and ethanol fractions of *Angelica keiskei* leaves and powder. Voucher specimens of the *Angelica keiskei* leaf and powder were deposited in the Laboratory of Food Enzyme Biotechnology, Kyungpook National University (#2010-Ak; Daegu, Korea).

### Skin irritancy test

In order to determine whether the *Angelica keiskei* fractions have toxic effects on the middle back skin of male 9-week-old NZW rabbits (2.1–2.4 kg), several toxicity parameters were evaluated. NZW rabbits are widely used for safety testing. Since a large amount of data for NZW rabbits has been accumulated over a long period of time, it is relatively simple to interpret data from experiments using these animals. The aqueous and ethanol fractions were solubilized in propylene glycol at a concentration of 10 mg/ml. Approximately 24 h prior to the administration of the test samples, the rabbit fur was carefully removed with an electric haircutter. The skin of the shaved back area was divided into four compartments (2.5×2.5 cm); two compartments served as the control areas and two were the test areas. Each compartment was diagonally located from its matching group member in the wound or non-wound group. In the wound group, each site was scratched with an 18-G needle so that only the epithelial tissues were damaged without drawing blood and a # symbol was scratched into the skin. The test sample was applied to each compartment of the skin on the back (90.5 ml/site) using 3-fold gauze (2.5×2.5 cm), then covered with squares of gauze (10×10 cm) and fixed with tape in order to prevent leakage and evaporation. The test substance was removed by carefully removing the gauze squares after 24 h. Draize skin reactions (20) were evaluated and scored by observing skin erythema, crust formation and edema following the administration of the test samples (24, 48 or 72 h). The average score was calculated by adding the scores for edema formation according to the primary skin irritation index (primary irritation index, PII) as shown in Table I.

### Phototoxicity test

A phototoxicity test was conducted using Hartley guinea pigs. The animals were divided as follows: untreated group, two experimental (aqueous and ethanol fractions) groups and a positive control group treated with 8-methoxypsoralen (8-MOP). Each group contained five guinea pigs (seven-week-old males weighing between 319.6 and 372.9 g). The untreated group was exposed to propylene glycol. For the two experimental groups, 0.5 ml/site of the aqueous or ethanol fraction were applied. The treated skin was then irradiated with ultraviolet (UV) light at a distance of 10 cm for 10 min using a UV irradiation apparatus (UVITEC LF-206. LS, Strasburg, France) with a UV lamp (365 nm). The left site was designated as the light irradiation site, whereas the right site was not irradiated. After 2, 4 and 24 h of irradiation, skin erythema, eschar and swelling was scored relative to the control. Transdermal administration was performed by removing the fur in a 4×6-cm area with an electric hair cutter and applying the test sample to two regions (each 2×2 cm). The test group samples were 0.5 ml at a concentration of 10 mg/ml, while 0.5 ml of a 0.1% solution of 8-MOP was applied at each side of the test site as a positive control (21). The non-irradiated site was shielded using aluminum tape.

### Analysis of parameters

Lesions were examined at 24, 48 and 72 h during the skin irritancy test and 2, 4 and 24 h after applying of the test fractions to evaluate phototoxicity. The designated criteria were strictly observed. Skin irritation and phototoxic effects were evaluated by measuring irritancy, edema or inflammation by trained examiners under the supervision of a veterinary pathologist from the Center for the Care and Use of Laboratory Animals, Kyungpook National University (Daegu, Korea).

## Results and discussion

In a previous study by our laboratory, it was determined that aqueous and ethanol fractions of *Angelica keiskei* leaf have potent whitening and anti-atopic activities at a concentration of 100 mg/ml (19).

The *Angelica keiskei* fractions were previously reported not to be toxic. During an eye irritancy test, no hazing, swelling, redness or emissions from the eye mucosa were observed and the fractions were revealed to have potential use in the cosmetic industry or other associated purposes (17). To first determine whether the fractions were cytotoxic, RAW264.7 cells were used for an *in vitro* assay. The results from this assay demonstrated that the aqueous and ethanol fractions were not cytotoxic to the cells at a concentration of 300 mg/ml (data not shown).

Currently, there are numerous plants used for industrial applications due to their beneficial properties. The *Angelica* sp. is a valuable herb used in Korea, Japan and other Far Eastern countries for its antioxidant and immunomodulatory activities (4). *Angelica* leaves contain various nutrients, including minerals, vitamins, flavonoids and other polyphenol compounds (8,9). This plant also has a significant potential for other purposes, including utilization as a cosmetic ingredient. To ensure the safe use of this and other ingredients, only animal data should be submitted, such as results from Draize eye mucosal irritancy, skin irritancy and phototoxicity tests. These tests involve applying reagents/substances to rabbit/guinea pig eyes or skin. When assessing the safety of the ingredients, guidelines for the use of a test material should be based on data from several skin toxicity tests.

Major factors correlated with toxicity are associated with amines, nitrous compounds or detrimental substances which may be produced during plant growth, storage, preservation, processing or cooking. However, there are a number of studies describing the antitumor, antidiabetic and anti-inflammatory activities of extracts from the *Angelica* sp. (2,3). To examine the biological activities of the *Angelica* sp., our group previously performed a study to determine whether these extracts had anti-atopic dermatitis or anti-asthmatic properties (22). These types of ingredients may be extracted from raw fruits, vegetables and medicinal herbs. Subsequently, the ingredients may be converted into more potent food biomaterials using various techniques, such as fermentation, biotransformation or chemical modification. The final products may be converted into a cosmetic, cosmeceutical, nutraceutical or pharmaceutical compound. To investigate this point we first performed tests to obtain a biological profile for the anti-melanogenesis and anti-asthmatic activity of *Angelica keiskei*(19,22).

**Figure 1:**
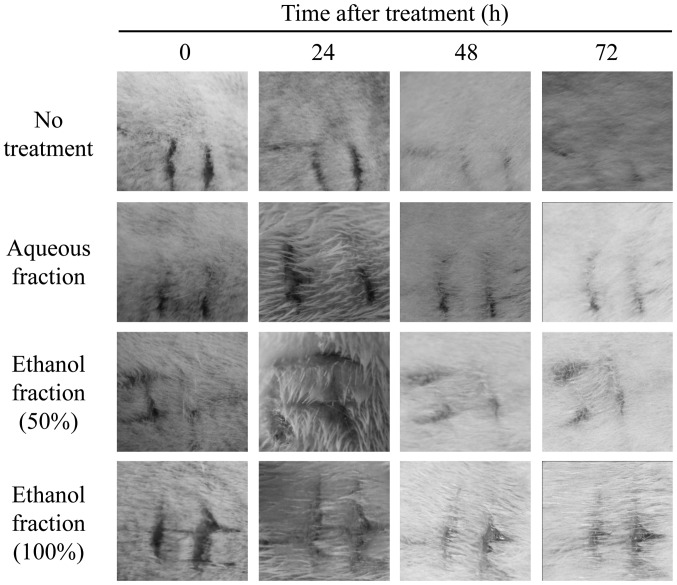
Comparison of skin irritancy test results measuring the effects of aqueous and ethanol fractions of *Angelica keiskei* leaves on skin with excoriation. A skin irritancy test was performed as described in Materials and methods.

**Figure 2:**
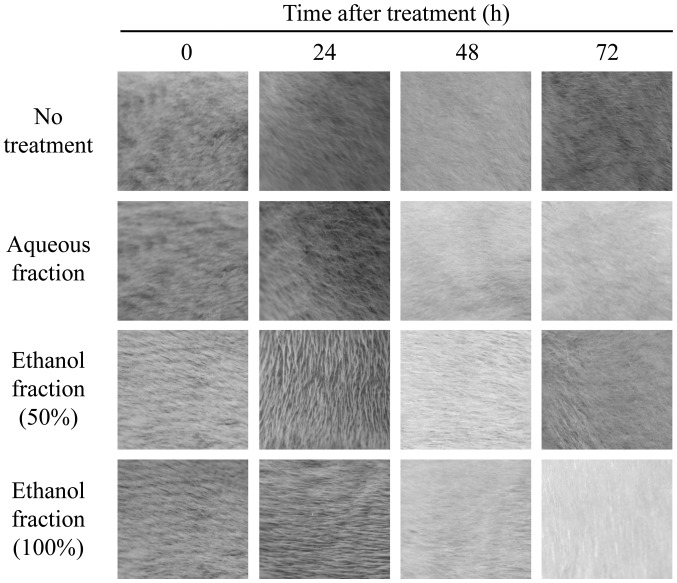
Comparison of skin irritancy test results measuring the effect of aqueous and ethanol fractions of *Angelica keiskei* leaves on skin without excoriation. Data were derived from three independent experiments.

In the present study, the skin irritancy potential of the *Angelica keiskei* leaf aqueous and ethanol fractions (100 mg/dose) was investigated by applying these compounds to the skin of rabbits. When the animal skin was wounded with an 18-G needle in the designated area, all skin symptoms appeared in the same pattern (Fig. 1). After 24, 48 and 72 h, wound healing proceeded naturally in the control group (Fig. 1, 1st row), while wound healing also progressed naturally in the test groups (2nd, 3rd and 4th rows). Since the Draize skin irritancy test strictly evaluates phenotypic characteristics and does not fully reflect the degree of skin irritancy, the toxicity test is considered imprecise and unreliable. However, this test is currently the most accurate for analyzing animal models. Therefore, the following two tests were used to accurately evaluate toxic symptoms with (Fig. 1) or without (Fig. 2) skin excoriation. Normally, wounds heal naturally via the homeostasis system of the body. To assess whether wound healing in skin treated with fractions was affected, the skin of the rabbits was damaged by inflicting abrasions and the healing process was monitored. Skin abrasions with or without the application of aqueous or ethanol fractions (Figs. 1 and 2) were examined over time and compared. The aqueous and ethanol (50% and 100%) fractions did not affect the skin damage (Fig. 1), suggesting that these fractions are not toxic. Similar results were also obtained from skin that had not been wounded (Fig. 2), indicating that the fractions tested have no effects on the skin. The final results demonstrated that the skin irritation index score was 0. Therefore, the findings indicate that neither the aqueous nor ethanol fraction irritated the skin, suggesting that the fractions caused no prick erythema or eschar on the skin and no additional symptoms.

**Figure 3:**
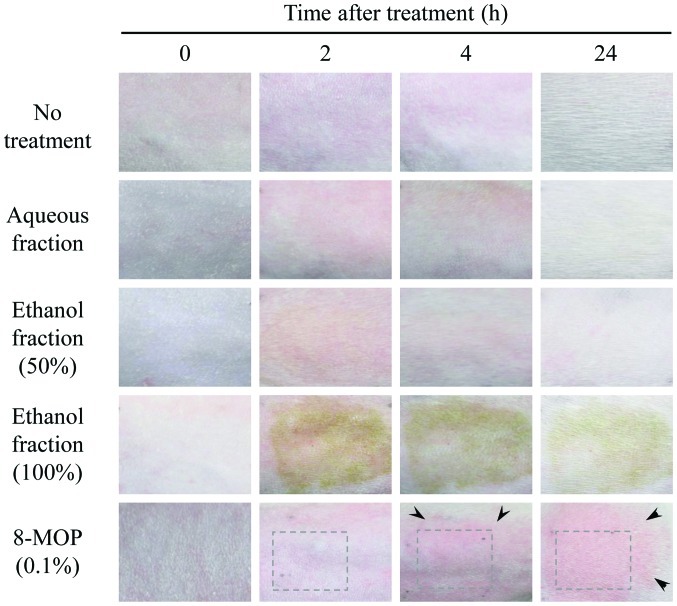
Comparison of phototoxicity test results measuring the effects of aqueous and ethanol fractions of *Angelica keiskei* leaves. Data shown are from a set of three independent experiments. The guinea pigs were housed in cages for one week. Images present each column of skin at different time intervals following treatment. Differences in skin color depended on exposure time of fractions or the positive control during image capturing. 8-MOP, 8-methoxypsoralen. Dotted boxes indicate that 8-MOP was applied to the area. Arrows denote phototoxic responses.

Phototoxicity was subsequently evaluated by analyzing skin exposed to UV irradiation. After removing the fur, guinea pig skin was treated with the aqueous and ethanol fractions and 8-MOP. In this test, the degree of erythema was determined on the following scale: 0 for no erythema, 1 for slight erythema, 2 for well-defined erythema, 3 for moderate to severe erythema and 4 for severe erythema to slight eschar formation. Similar erythema symptoms were observed up to 4 h after UV irradiation. After 24 h, the fraction-treated groups exhibited no symptoms of toxicity in the skin, unlike the positive control. Treatment with the fractions did not lead to erythema or eschar, while 8-MOP (0.1% as a positive control) caused moderate to severe erythema (Fig. 3). To measure edema, the following scale was used: 0 for no edema, 1 for slight edema, 2 for well-defined edema, 3 for moderate to severe edema and 4 for severe edema. The results demonstrated that the fractions did not cause erythema or eschar, whereas 8-MOP administration resulted in slight edema (Fig. 3). The final score was determined by assessing the average of the total values of erythema, edema and crust formation as follows: 0.0–0.5 for almost no phototoxicity, 0.6–1.2 for slightly phototoxic, 1.3–2.5 for clearly and highly phototoxic and 2.6–5.0 for severely phototoxic. As shown in Table I, all three samples (aqueous, 50% ethanol and 100% ethanol fractions) had scores of only 0.0–0.5, suggesting that the fractions tested in the experiment were non-irritating. However, 8-MOP was observed to be an irritating compound that caused erythema, eschar and edema (Fig. 3). Following 2 to 4 h of UV irradiation, slight redness was observed in all fraction-treated groups but this redness disappeared after 24 h. By contrast, groups treated with 8-MOP developed erythema and edema, indicating that the results of the phototoxicity test were achieved normally. Taken together, the findings suggest that while 8-MOP induced erythema, edema and/or eschar, no experimental samples caused moderate to severe toxicity. Based on this information, we determined that all fractions tested (aqueous, 50% ethanol and 100% ethanol fractions) did not exhibit skin irritation caused by UV irradiation, whereas 8-MOP was associated with toxicity which caused skin irritation.

In conclusion, the present study investigated whether fractions of *Angelica keiskei* extract potentially cause skin irritation and phototoxicity. None of the fractions was found to irritate the skin or to be phototoxic, indicating that these fractions may be useful in the cosmetic or cosmeceutical industry and for other applications. Although the fractions were derived from an edible plant, their potential phototoxicity requires further evaluation and additional skin irritancy tests must be performed.

## Figures and Tables

**Table I:** Total scores from the phototoxicity test evaluating the effects of aqueous and ethanol fractions obtained from *Angelica keiskei* leaves.

Criteria	Total scores	Aqueous	50% Ethanol	100% Ethanol	0.1% 8-MOP
Non-irritating	0.0–0.5	Yes	Yes	Yes	-
Minimally irritating	0.6–1.2	-	-	-	-
Severely irritating	1.3–2.5	-	-	-	Yes
Extremely irritating	2.6–5.0	-	-	-	-

8-MOP, 8-methoxypsoralen.
